# A Novel *In Vitro* Multiple-Stress Dormancy Model for *Mycobacterium tuberculosis* Generates a Lipid-Loaded, Drug-Tolerant, Dormant Pathogen

**DOI:** 10.1371/journal.pone.0006077

**Published:** 2009-06-29

**Authors:** Chirajyoti Deb, Chang-Muk Lee, Vinod S. Dubey, Jaiyanth Daniel, Bassam Abomoelak, Tatiana D. Sirakova, Santosh Pawar, Linda Rogers, Pappachan E. Kolattukudy

**Affiliations:** Burnett School of Biomedical Sciences, College of Medicine, University of Central Florida, Orlando, Florida, United States of America; University of Hyderabad, India

## Abstract

**Background:**

*Mycobacterium tuberculosis* (*Mtb*) becomes dormant and phenotypically drug resistant when it encounters multiple stresses within the host. Inability of currently available drugs to kill latent *Mtb* is a major impediment to curing and possibly eradicating tuberculosis (TB). Most *in vitro* dormancy models, using single stress factors, fail to generate a truly dormant *Mtb* population. An *in vitro* model that generates truly dormant *Mtb* cells is needed to elucidate the metabolic requirements that allow *Mtb* to successfully go through dormancy, identify new drug targets, and to screen drug candidates to discover novel drugs that can kill dormant pathogen.

**Methodology/Principal Findings:**

We developed a novel *in vitro* multiple-stress dormancy model for *Mtb* by applying combined stresses of low oxygen (5%), high CO_2_ (10%), low nutrient (10% Dubos medium) and acidic pH (5.0), conditions *Mtb* is thought to encounter in the host. Under this condition, *Mtb* stopped replicating, lost acid-fastness, accumulated triacylglycerol (TG) and wax ester (WE), and concomitantly acquired phenotypic antibiotic-resistance. Putative neutral lipid biosynthetic genes were up-regulated. These genes may serve as potential targets for new antilatency drugs. The triacylglycerol synthase1 (*tgs1*) deletion mutant, with impaired ability to accumulate TG, exhibited a lesser degree of antibiotic tolerance and complementation restored antibiotic tolerance. Transcriptome analysis with microarray revealed the achievement of dormant state showing repression of energy generation, transcription and translation machineries and induction of stress-responsive genes. We adapted this model for drug screening using the Alamar Blue dye to quantify the antibiotic tolerant dormant cells.

**Conclusions/Significance:**

The new *in vitro* multiple stress dormancy model efficiently generates *Mtb* cells meeting all criteria of dormancy, and this method is adaptable to high-throughput screening for drugs that can kill dormant *Mtb*. A critical link between storage-lipid accumulation and development of phenotypic drug-resistance in *Mtb* was established. Storage lipid biosynthetic genes may be appropriate targets for novel drugs that can kill latent *Mtb*.

## Introduction

One third of the world population is carrying latent TB infection [1], [2]. The ability of the pathogen to go into the phenotypically drug-resistant non-replicating dormant state in such latent infection is a major impediment to curing the disease since currently available drugs cannot kill latent *Mtb*. The emergence and spread of multidrug-resistant (MDR) or extremely drug resistant (XDR) TB complicates this problem especially with the spread of AIDS world wide [2], [3]. Development of drugs that can kill phenotypically drug-resistant *Mtb* in patients with latent TB infection is an extremely urgent need. The development of antibiotic resistance in non-replicating dormant bacteria which is described as ‘phenotypic drug-resistance’ or ‘drug-tolerance’ is due to changes in the physiological state of the bacteria and not conferred by any inheritable genetic resistance mechanism [4]. Typically, the phenotypic drug-resistance of dormant *Mtb* is exemplified by resistance to the sterilizing antibiotic rifampicin (Rif) and is regarded as one of the hallmarks of latent TB. Several animal models of latent TB have been developed [5]. However, it is unlikely that any of them truly represent the human latent TB [5], [6]. *In vitro* models of latent *Mtb* suitable for screening chemical libraries to discover drugs that can kill latent *Mtb* are not available.

Metabolic processes that are critical for the pathogen to go into dormancy, survive under this non-replicating drug-resistant state, and get reactivated when the immune system of the host is weakened remain poorly understood. It has been recognized for more than half a century that the pathogen inside the host utilizes fatty acids as the major energy source and that glyoxylate cycle plays a critical role in the use of fatty acids as the main carbon source [7], [8]. Convincing evidence obtained in recent years have shown that the glyoxylate cycle plays a critical role in the persistence of the pathogen in the host [9], [10]. However, the origin of the fatty acids and the nature of the storage form of fatty acids are not clear [9], [11]. We have shown that the pathogen stores energy as triacylglycerol (TG) as it goes into dormancy-like state *in vitro* and uses this stored energy to survive during starvation [12]–[14]. We have also reported the functional characterization of products of 15 triacylglycerol synthase (*tgs*) genes and identified *tgs1* product as the dominant contributor to storage of TG that occur when *Mtb* is exposed to different single stress factors [12], [14].

Many organisms use wax esters (WE) as the major form of energy storage [15]. For example, seeds of some plants, such as jojoba, and many marine organisms use WE as the major form of energy storage. TG and WE are important storage lipids in some groups of prokaryotes [16]. Nothing is known about the enzymatic biosynthesis of WE in mycobacterial species. Based on our earlier observation that some of the mycobacterial *tgs* gene products expressed in *E. coli* showed significant activity for WE synthesis [12], we speculated that some of these *tgs* genes may play a role in biosynthesis and accumulation of WE under multiple-stress condition. Enzymatic steps involved in the biosynthesis of WE were first elucidated in our laboratory [17], [18]. More recently the enzymatic strategy used in the production of alcohol used in WE biosynthesis was elucidated [19]. We identified three putative fatty-acyl-CoA reductase (*fcr*) genes in *Mtb* genome and measured their expression under the multiple-stress condition.

Several studies have explored possible stress conditions that *Mtb* would face in the host where the pathogen goes into the latent state [20]–[24]. In attempts to elucidate gene expression changes that occur as the pathogen goes into a dormant state, the pathogen has been subjected to certain stress factors thought to be encountered by the pathogen in the host. Such stress factors so far tested include hypoxia (slow oxygen depletion), nutrient deprivation, NO treatment and growth in acidic media [21]–[29]. There were considerable variations in gene expression profile changes under such individual stress conditions [30]. Some conditions caused accumulation of storage lipids while others made the pathogen resistant against a low concentration of Rif [12], [24], but a critical link between metabolic requirement and dormancy phenotype development has not been established. There are reports that bacilli within tuberculous lesions like the granuloma or closed cavity, encounter low oxygen but not severe hypoxia and high CO_2_ concentrations along with low nutrient levels and acidic condition [21], [31]–[34]. Therefore in an attempt to mimic the conditions that the pathogen might encounter in the host, we applied a combination of these four stresses (multiple stress) on *Mtb* in culture.

Our experimental results show that the application of these four stress factors in combination led to the accumulation of TG and WE in *Mtb* cells and these cells lost acid-fastness. The genes involved in the biosynthesis of these storage lipids were induced during the development of dormancy-like features under multiple-stress. Up-regulation of known stress-responsive regulatory genes also implicated the achievement of dormant state. Lipid accumulation and phenotypic Rif- and isoniazid (INH)-resistance increased in *Mtb* population under multiple-stress. The *tgs1* deletion mutant, that showed an inability to accumulate TG, was not able to develop antibiotic tolerance at the level of the wild type strain and complementation restored antibiotic tolerance. Thus we were able to demonstrate a link between storage-lipid accumulation and development of drug-tolerance in the *Mtb* cells during the development of dormancy. We have shown that with a redox indicator dye, Alamar Blue, we can measure the viable *Mtb* cell count remaining after treatment with antituberculosis drugs. Adaptation of this method would allow high-throughput screening for drugs that can kill dormant *Mtb*.

## Results

### *Mtb* accumulates storage lipids under multiple-stress

**Figure 1:**
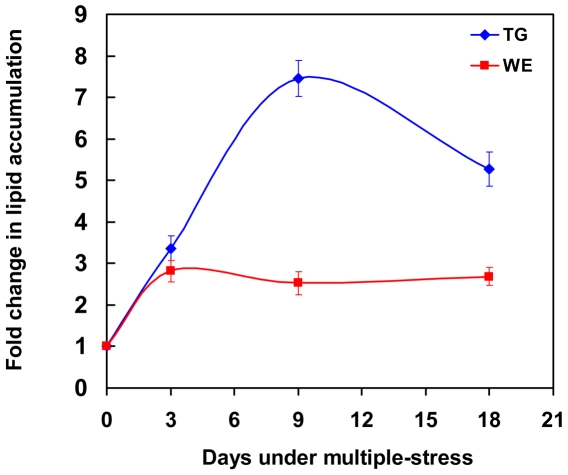
Accumulation of storage lipids by *Mtb* under multiple-stress *in vitro*. *Mtb* was subjected to a combination of four stresses - high CO_2_, low O_2_, acidic pH and nutrient starvation. Total lipids were extracted at 0, 3, 9 and 18 days and resolved on silica-TLC using hexane-diethyl ether-formic acid (90∶10∶1, v/v/v). Lipids were visualized by charring after spraying with dichromate-sulfuric acid and quantified by densitometry using Alpha Innotech Gel documentation system and AlphaImager 2200 software (Alpha Innotech, USA). TG, triglycerides; WE, wax esters.

**Figure 2:**
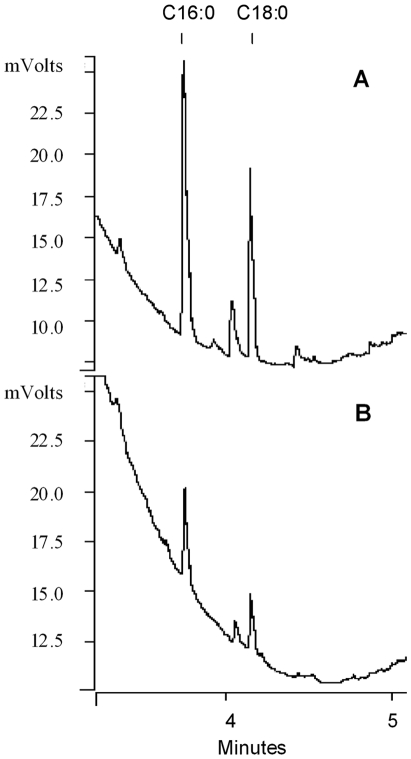
WE and TG accumulated by *Mtb* under multiple-stress is composed mainly of C_16:0_ and C_18:0_ fatty acids. WE (A) and TG (B) accumulated by *Mtb* after 18 days under MS were trans-esterified and analyzed by capillary gas chromatography on Varian CP-TAP CB column using a temperature program as described in Materials and Methods.

Since the hypoxic or NO-stress conditions caused accumulation of storage lipids but did not result in detectable Rif-resistance against moderate concentrations of Rif (5 µg/ml) (unpublished results, [24], [35],), an important indicator of true dormancy, we tested a combination of stress conditions which the pathogen is thought to encounter in the host [10], [21], [31], [33], [34]. After growing *Mtb* in complete Dubos medium (OD_600 nm_ = 0.2) the cells were transferred to a low-nutrient medium (10% Dubos medium without glycerol) at acidic pH (pH 5.0) in an atmosphere containing high (10%) CO_2_ and low (5%) O_2_ for 18 days and the lipid accumulation in those *Mtb* cells was analyzed at 3, 9 and 18 days. We found that TG accumulation reached maximal level by day 9 (Fig. 1). WE levels in *Mtb* cells also increased significantly and reached maximal levels by 3 days (Fig. 1). The aerobic control samples at pH 5.0 or 7.0 did not show an increase in WE or TG (data not shown). Capillary-GC analysis revealed that palmitate (C_16:0_) and stearate (C_18:0_) were the major fatty acid constituents of the TG and WE fractions (Fig 2). Metabolic incorporation of ^14^C-oleic acid into lipids by the *Mtb* cells after 0, 9 and 18 days under multiple-stress revealed that ^14^C was incorporated predominantly into TG, and polar lipids (Table 1). Incorporation of ^14^C-oleic acid into TG increased from 12% of the label in total lipids on day 0 to 43% on day 9 decreasing slightly to 37% on day 18. Incorporation of the radiolabeled oleic acid into polar lipids decreased from 51% on day 0 to 17% on day 9 reflecting down regulation of the biosynthesis of membrane lipids and channeling of fatty acids into storage lipids as the pathogen goes into the dormant state. Incorporation of ^14^C-oleic acid into WE increased significantly on day 9 and remained at the same level at day 18 (Table 1).

**Table 1:** *Mtb* cells under multiple-stress incorporate [^14^C]oleic acid primarily into triglycerides.

Days under multiple-stress	Radioactivity as Percent of Total Lipids in		
	Triglycerides	Wax Esters	Polar lipids
0	12	0.8	51
9	43	1.6	17
18	37	1.5	20

At 0, 9 or 18 days under multiple-stress, *Mtb* cultures were metabolically labeled with [1-^14^C]oleic acid for 6 h and total lipids were extracted and resolved on TLC as described previously [12] using hexane∶diethyl ether∶formic acid (80∶20∶2, v/v/v) as the solvent system. Regions of the TLC plates corresponding to standard triolein (for triglycerides) or oleyl oleate (for wax esters) or the origin (for polar lipids) were scraped and radioactivity was quantified by liquid scintillation counting. Radioactivity in individual lipid subclasses is expressed as a fraction of the radioactivity in total lipid extracts prior to TLC procedures.

### Development of phenotypic Rif- and INH-resistance by *Mtb* under multiple-stress

Multiple stresses inside the host are thought to cause *Mtb* to go into a non-replicating, drug-tolerant dormant state [10], [21], [31], [33], [34]. We found that when *Mtb* was subjected to the combination of multiple stresses *in vitro*, it developed higher degree of phenotypic resistance to Rif and INH indicating that a higher percentage of the mycobacterial population entered a dormant state. Nearly the entire *Mtb* population at the beginning of the multiple-stress treatment (day 0) was killed (0.005 to 0.03% survival) by both Rif (5 µg/ml) and INH (0.8 µg/ml). By day 9 under multiple-stress, phenotypic resistance of *Mtb* cells against Rif (5 µg/ml) increased by about 120-fold (∼5% survival) and resistance against INH (0.8 µg/ml) increased by about 1200-fold (∼35% survival) (Table 2). After 18 days under multiple-stress, the resistance of *Mtb* cells to Rif and INH were about 310-fold (∼12% survival) and 2800-fold (∼84% survival), respectively, compared to the survival level at day 0 (Table 2). At a lower concentration of Rif (0.1 µg/ml), which was used in the slow oxygen depletion model developed by Wayne and Hayes [24], 100% of the *Mtb* population was resistant at both 9 and 18 day time periods under multiple-stress (data not shown). Up to about 50% of the *Mtb* population was resistant to Rif at 1 µg/ml after 18 days under the combined stresses (data not shown). This degree of Rif-resistance developed in the *Mtb* cultures under multiple-stress condition is dramatically higher than that obtained in the hypoxia model of Wayne and Hayes [24].

**Table 2:** Development of Rif-resistance in wild type *Mtb* H37Rv but not in Δ-*tgs1* mutant upon application of multiple-stress; complementation restores Rif-resistance.

*Mtb* strains	Days under Multiple-stress	Resistance to Antibiotics (%)	
		INH (0.8 µg/ml)	Rif (5.0 µg/ml)
WT-H37Rv	0	0.03 (±0.01)	0.04 (±0.02)
	9	34.7 (±12)	4.7 (±1.9)
	18	84.4 (±17.5)	12.5 (±3.4)
Δ-*tgs1*	0	0.01 (±0.01)	0.03 (±0.02)
	9	21.1 (±7.8)	1.2 (±0.9)
	18	31.2 (±13.1)	1.9 (±0.9)
C-Δ-*tgs1*	0	0.04 (±0.02)	0.03 (±0.01)
	9	37.9 (±13.5)	5.2 (±2.1)
	18	91.0 (±19)	11.0 (±4.5)

*Mtb* cultures at 0, 9 or 18 days of multiple-stress were treated with Rif (5 µg/ml) or INH (0.8 µg/ml) for 5 days. Viable bacilli were enumerated by the cfu count method and compared with controls not subjected to antibiotic treatment. WT-H37RV, Mtb H37RV, Δ-*tgs1*, *tgs1* deletion mutant of WT-H37Rv, C-Δ-*tgs1*, complemented *tgs1* mutant. Rif, Rifampicin; INH, Isoniazid.

### Loss of acid-fastness and accumulation of lipid bodies by *Mtb* under multiple-stress

**Figure 3:**
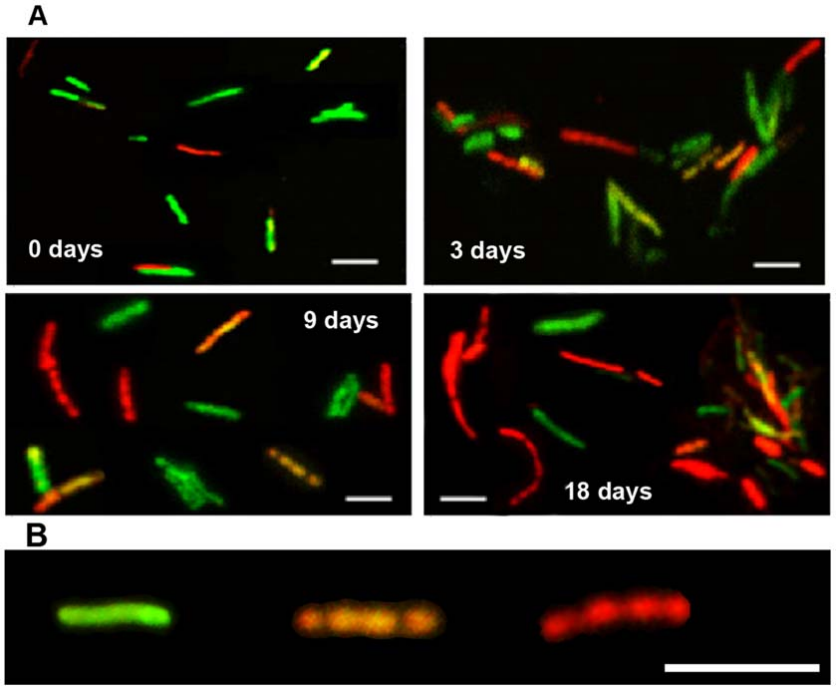
Accumulation of lipid bodies and loss of acid-fastness in *Mtb* cells under multiple-stress. (A), Acid-fast staining cells (green) decreased and lipid body staining cells (red) increased with time under multiple-stress. Cells were stained with Auramine-O (acid-fast stain) and Nile Red (neutral lipid stain) and examined by confocal laser scanning microscopy (Leica TCS SP5) at the same laser intensity for all the samples with Z-stacking to get the depth of the scan field. Scanned samples were analyzed by LAS AF software for image projection. Overlaid images of the dual-stained *Mtb* are shown. Bar = 4 µM. (B), Magnified view of three different *Mtb* cells, representing three different subsets of *Mtb* cells in terms of acid-fast and neutral lipid staining property, observed in the *Mtb* population under multiple-stress: only acid-fast positive without any Nile Red stain (green), both acid-fast and lipid stain positive (orange yellow) and acid-fast negative cells with only Nile Red staining lipid bodies (right). The only acid-fast stain (green) positive cells gradually decreased and the other two types steadily increased during multiple-stress treatment. These cells selected from a day 9 sample were stained with both dyes and examined by confocal scanning as stated above in (A). Bar = 5 µM.

**Figure 4:**
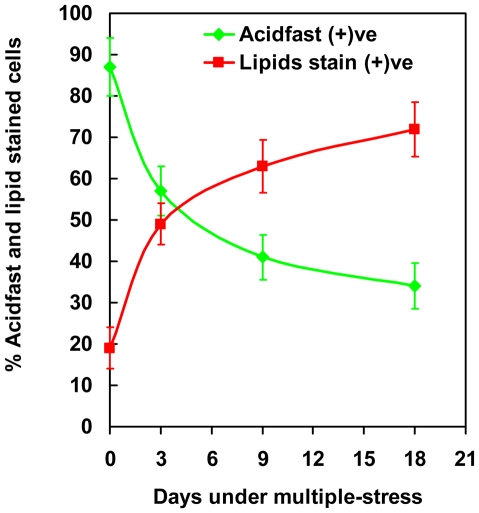
Increase in the percentage of lipid-stained cells and decrease in the percentage of acid-fast stained cells in *Mtb* culture subjected to *in vitro* multiple-stress. Number of Auramine-O stained acid-fast positive (green) and Nile Red stained lipid body positive cells (red) were counted from multiple microscopic scans as presented in the figure 3.

Dual staining of *Mtb* with the combination of Auramine-O and Nile Red has been used to reveal acid-fast staining property and neutral lipid accumulation in the same cell [36]. We applied this dual staining procedure to examine acid-fastness of *Mtb* cells and lipid body accumulation within the *Mtb* cells under multiple-stress. When we subjected a young, synchronous culture of *Mtb* to the multiple-stress condition for increasing periods of time, we observed a steady decrease in Auramine-O stained green-fluorescing acid-fast cells with a corresponding increase in Nile Red stained red-fluorescing lipid-body containing cells (Fig. 3). Initially, in the freshly grown starter culture about 90% of the population was acid-fast positive by retaining the green-fluorescing Auramine-O stain, and only a few lipid-body accumulating (Nile Red) cells could be detected. Most of these Nile Red positive cells also retained the acid-fast specific Auramine-O stain in the heterogeneous population at day 0. After 18 days under multiple-stress, acid-fast positive cells decreased to about 30% of the population while Nile Red-stained cells with internal red spherical bodies increased from 10% to about 70% (Fig. 4). Overlaid green and red color images of dual-stained *Mtb* showed some cells staining with both Auramine-O and Nile Red and fluoresced at both green and red wavelengths to give an orange appearance while other cells stained exclusively for Auramine-O or Nile Red (Fig. 3). This difference in dual staining property indicated generation of at least three different sub-populations in the *Mtb* cultures under multiple-stress condition: a subset that stained only with Auramine-O: (probably actively multiplying), a second subset which stained with both Auramine-O and Nile Red (probably transitioning to non-replicating state) and a third subset that stained only with Nile Red (probably non-replicating and dormant) (Fig. 3B).

### *Mtb* cells become more buoyant when subjected to multiple-stress

**Figure 5:**
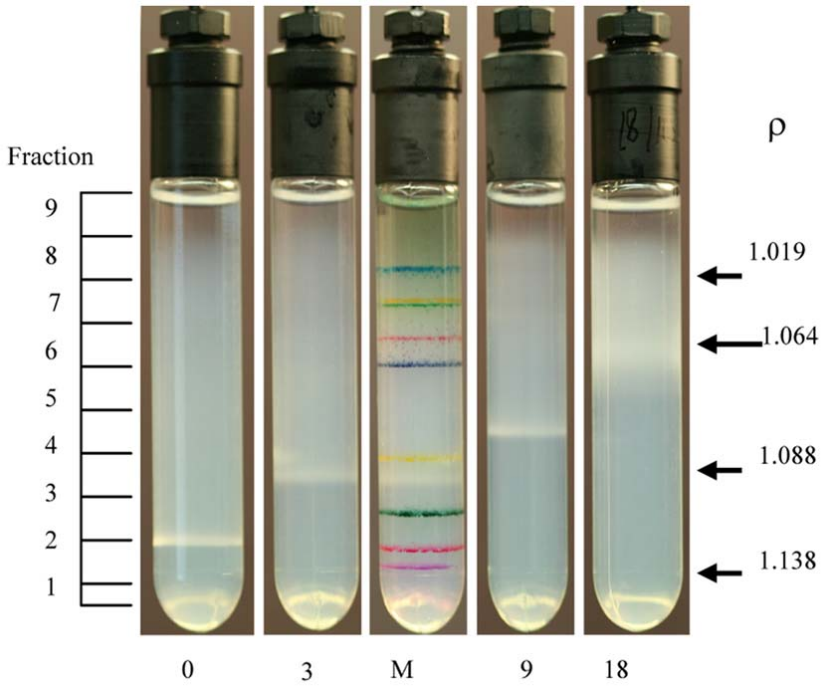
Decrease in buoyant density of *Mtb* cells subjected to multiple-stress. *Mtb* cells subjected to the multiple-stresses were placed on the preformed gradient and centrifuged at 400 g for 20 min. The center tube is a 3 day cell sample mixed with density marker (M) beads. Percoll gradients were self-formed by centrifugation from a starting solution with a density (ρ) of 1.0925 gm/ml. The densities of selected bead layers (ρ, in gm/ml) are given on the right, and the positions of one ml fractions collected for analyses are at the left. Numbers below the tubes indicate the number of days under multiple-stress.

**Figure 6:**
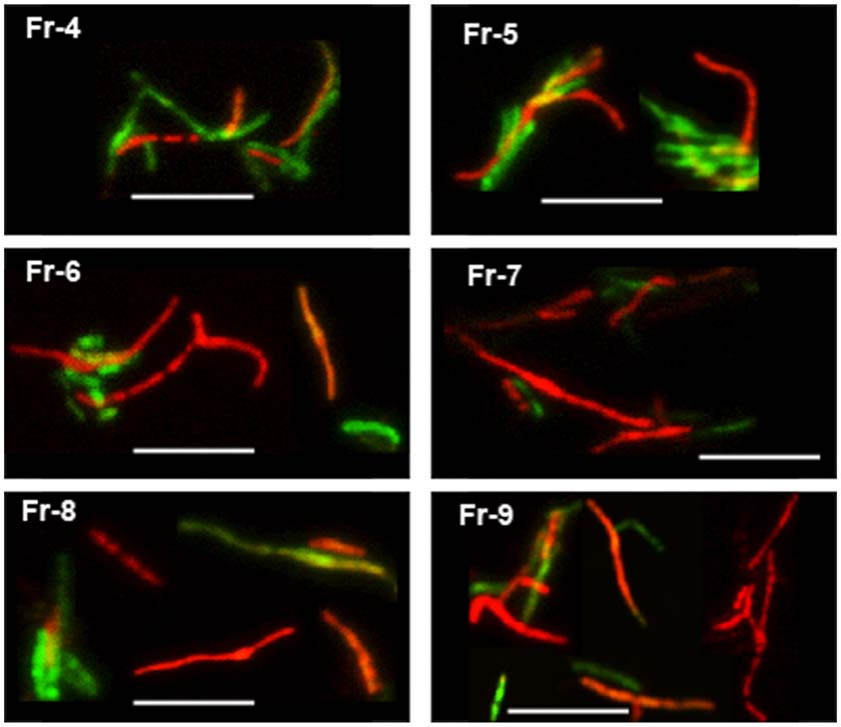
Auramine-O (green) and Nile Red staining of *Mtb* cells in Percoll gradient fractions of *Mtb* culture after 18 day in multiple-stress. Density gradient fractionation was performed as described in figure 5. Changes in acid-fast property, lipid accumulation and elongated cells with cording were observed. Nile Red staining *Mtb* cells concentrated in higher fraction numbers at lighter density. No cells were detected in fractions 1, 2 and only a few were detected in fraction 3. Fr, fraction; fraction numbers ascending from the bottom of the tube to the top. Bar = 5 µM.

We investigated whether the lipid accumulation in *Mtb* cells might be reflected in changes of buoyant density. *Mtb* cultures subjected to multiple-stress for different periods of time were fractionated on a Percoll density gradient and subsets of *Mtb* population were separated at different density levels at different time periods during multiple-stress treatment (Fig. 5). With increasing incubation time under multiple-stress, the bands of floating *Mtb* cells representing the major fraction of the *Mtb* population shifted towards lower buoyant density regions of the gradient in the upper phase of the tube (Fig. 5). This reflected the increase in lipid-loaded and non acid-fast-staining cells in the population with time under multiple-stress. Percoll density gradient fractionation of the *Mtb* culture, after 18 days under multiple-stress, showed a population of cells as a diffuse band in the lighter density region and the great majority of the lipid-droplet containing cells were distributed in the lighter fractions (Fig. 5; fractions 6, 7 and 8). Auramine-O/Nile Red staining of the different fractions showed that with increasing periods under the multiple-stress condition, increasing number of cells became lipid-loaded and lost acid-fast staining property. Nile Red staining of Percoll density gradient fractions from 18 day-stressed cultures showed that the lighter fractions were more enriched in lipid-loaded cells that lost acid-fastness (Fig. 6). These changes are consistent with the conclusion that application of multiple-stress caused progressive changes in lipid accumulation resulting in increasing percentages of presumably dormant cells in the lighter fractions.

### Transcriptomic profile of *Mtb* cells indicates down-regulation of genes involved in energy metabolism, transcription and translation, and up-regulation of stress responsive genes under multiple-stress

**Figure 7:**
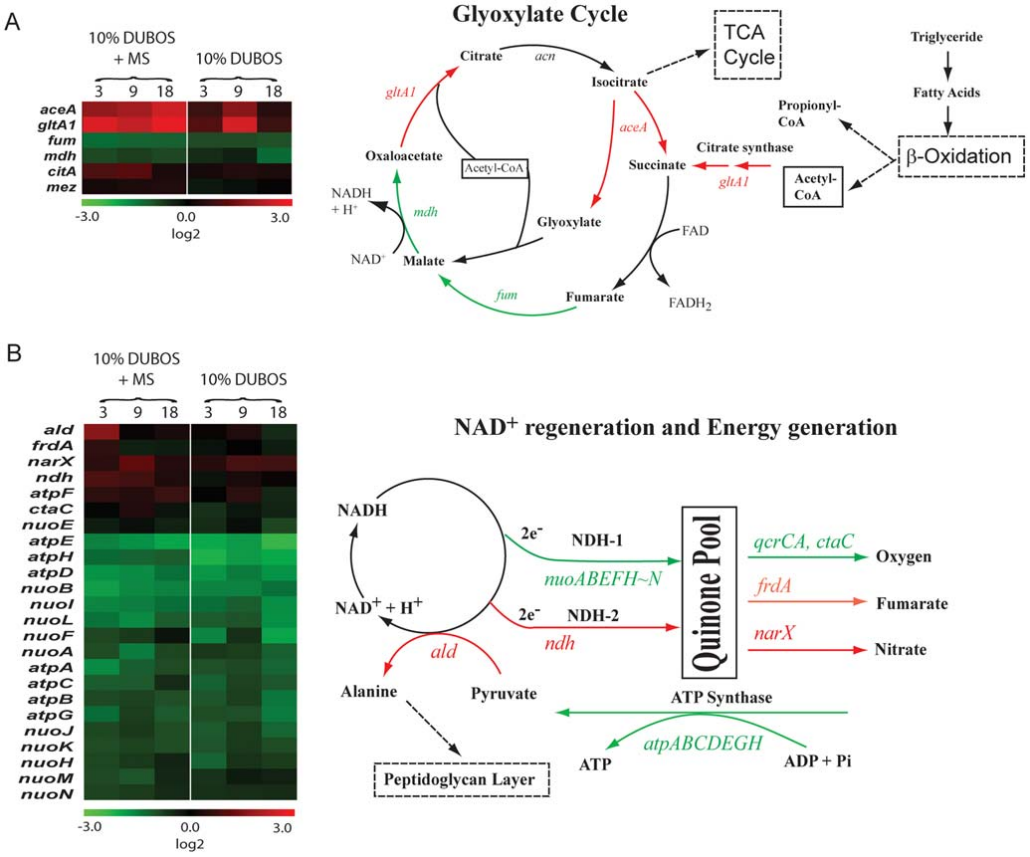
Microarray analysis demonstrated changes in expression of genes involved in glyoxylate cycle and energy metabolism. (A), The expression ratio of genes involved in glyoxylate shunt cycle was shown in the red-green-display according to the log2-tranformed color code. Experimental time-points were shown at the top of the column. Genes were selected based on their annotation in TubercuList database, and grouped into those that were either regulated at least one of the time-points under multiple- stress condition. (B), Energy generation and NAD regeneration under multiple-stress. Genes involved in energy generation were selected based on their annotation. Red denotes induction and green denotes repression.

We analyzed changes in the gene expression profiles using oligonucleotide microarray. Analysis of variance (ANOVA) and significance analysis of microarrays (SAM) [37] were conducted to identify significant gene expression changes at selected time points during the 18 days of multiple-stress application to *Mtb* cells. The genes that were differentially expressed more than 2-fold include a total of 331 targets, representing ∼7% of ORFs on the chip. Under the multiple-stress condition, genes that encode enzymes of glyoxylate cycle such as isocitrate lyase (*icl* or *aceA/*Rv0467) and citrate synthase (*gltA1/*Rv1131c) showed significant increase in expression for all the time points examined (Fig. 7A). *Mtb* showed shutdown of both ATP and NAD energy regeneration systems (Fig. 7B). All the genes encoding NADH dehydrogenase I subunits (*nuoABEFHIJKLMN*) and ubiquinol–cytochrome C complex (*qcrA/B/C*) were repressed. In addition, the gene expression of ATP synthase subunits was repressed, indicating the major shutdown of ATP generation in the cells. Moreover, slowdown of the overall activity in transcription/translation apparatus was manifested under the multiple-stresses. For instance, *rhlE* (ATP-dependent RNA helicase homolog) was repressed, demonstrating reduced activity in transcription machineries. However, genes required for anaerobic respiration (*frdA*, *narG/H/X*, *nirA*) were continuously expressed until the later time points examined (18 days) and aerobic respiration was significantly repressed throughout the period of multiple-stress treatment. We also found significant induction of the genes classified as the stress-response genes (e.g. *hspX*/*acr*; Rv2031c) that may play a role in maintaining long term survival within the host [38].

### Functional clustering revealed nutrient starvation made a major contribution to changes in gene expression

**Figure 8:**
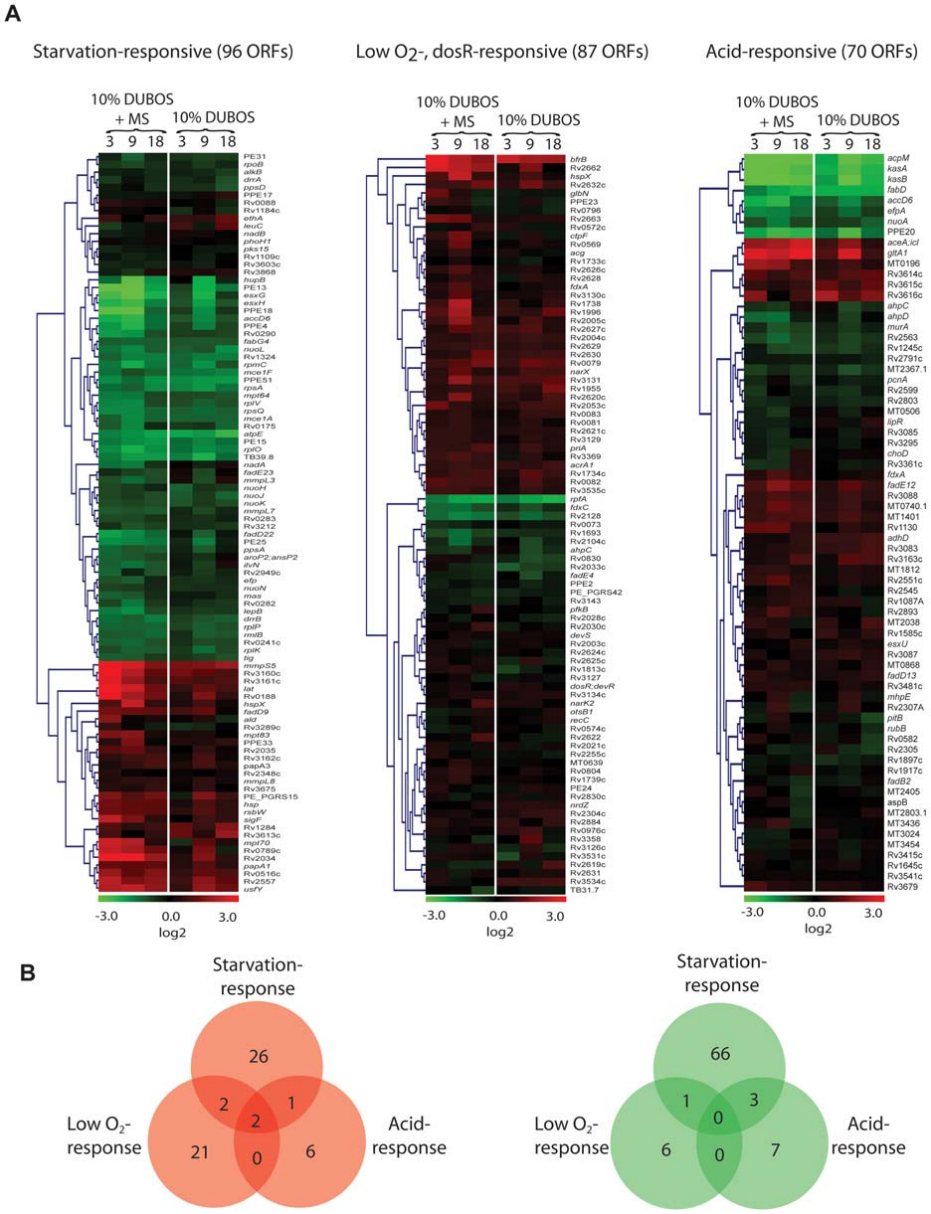
Functional clustering of *Mtb* genes under multiple-stress revealed nutrient starvation made major contribution to the number of genes that show changes in expression. (A), Microarray data of *Mtb* gene expression under multiple-stress compared to the respective expression data for selected genes reported for nutrient-starvation- [21], hypoxic- [39], [66], and low pH-response [22]. Expression ratios were averaged, log2-transformed, and displayed according to the color code at the bottom of the each column. Experimental time-points were indicated at the top of the each column. The Euclidean average linkage clustering (standard z-transformed) was performed to generate gene trees shown at the left side of each column. MS, Multiple-stress. (B), Venn diagrams showing the number of overlapping and unique set of genes modulated more than 1.8-fold at any time-points under multiple-stress condition. Induced or repressed genes were selected to categorize based on stress-response in red circle or green circle to indicate gene induction or repression, respectively.

Comparison of the transcription profiles of *Mtb* in this new multiple-stress model with those reported previously using single-stress applications such as nutrient depletion [21], acidic shock [22] and hypoxia [39] indicates that nutrient starvation caused changes in expression of larger number of gene than the other stresses did. Using transcription profile data from these reports, we were able to cluster the gene-expression data of *Mtb* under multiple-stress (Fig. 8A). This functional clustering was also verified using genes that changed their expression by more than 1.8-fold at any time point under multiple-stress. A total of 141 genes classified by their stress responsiveness were clustered and represented as Venn diagrams (Fig. 8B). Significantly induced genes were mostly placed in either starvation-responsive or low-oxygen-responsive gene clusters, while the repressed genes were mostly grouped in the starvation-responsive gene cluster. The number of genes repressed by nutrient-starvation was more than double the number of induced genes (26 vs. 66 genes; Fig. 8B).

### Real-time Taqman RT-PCR showed upregulation of storage lipid biosynthetic and dormancy-associated genes in *Mtb*

**Figure 9:**
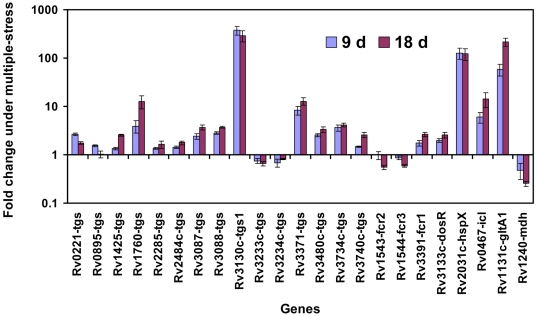
Real-time Taqman RT-PCR measurement of transcripts levels of selected genes potentially involved in dormancy and synthesis of storage lipids (TG and WE) in *Mtb* H37Rv under multiple-stress. Relative quantitation method (ddCt) was used with the 7500 Fast real time system and analysis was done using SDS v1.4 software of Applied Biosystems Inc. *sigA* was used as the endogenous control to normalize expression values and samples of starter cultures (day 0) were used as calibrator to calculate the fold induction. Y axis is in log scale.

Among the 15 *tgs* genes tested for their transcript levels in *Mtb* cells, upregulation of *tgs1* (Rv3130c) was the highest with about 370 and 300 fold induction at 9 and 18 days respectively under the multiple-stress condition (Fig. 9). Rv3371 and Rv1760 were also significantly induced by approximately 8 and 4 fold, respectively at 9 day and by about 12 fold at 18 day. Their expression levels increased significantly during the later time points, whereas induction of *tgs1* was maintained almost at the same level at both 9 and 18 days of multiple-stress treatment (Fig. 9). Among the remaining 12 *tgs* genes, 10 *tgs* genes have been found to be consistently induced at different levels, whereas Rv3233c and Rv3234c were found to be repressed at all the time points (Fig. 9). Induction of Rv3088 (*tgs4*), Rv3087, Rv3734c (*tgs2*) and Rv3480c were in the range of 2 to 4 fold compared to the starter culture and their induction level was higher on day 18 than on day 9 (Fig. 9). Rv0221, Rv1425, Rv2484c and Rv2285 genes were found to be induced in the range of >1.2 to ∼2 fold either on 9 or on 18 days under multiples-stress. In addition to increased TG accumulation, we have also detected increased accumulation of WE as another storage-lipid in the *Mtb* cells under multiple-stress. Three putative fatty-acyl-CoA reductase (*fcr*) genes (Rv3391/*fcr1*; Rv1543/fcr2; Rv1544/fcr3), which may take part in WE biosynthesis, were tested for their change in expression. Among the three putative *fcr* genes only Rv3391 (*fcr1*) was found to be up-regulated (Fig. 9).

To confirm the achievement of non-replicating dormant state in the *Mtb* cells under multiple-stress, we have also determined the expression pattern of a few known dormancy and stress responsive genes in each sample. Genes encoding glyoxylate shunt pathway enzymes, isocitrate lyase (*icl*/Rv0467) and citrate synthase (*glt*A1/Rv1131c) were found to be up-regulated by 14 and 216 fold, respectively after 18 days, whereas, malate dehydrogenase gene (*mdh*/Rv1240) was found to be consistently repressed up to 18 days under multiple-stress condition (Fig. 9). Stress responsive gene *hspX* (*acr*/Rv2031c) was up-regulated by approximately 120 fold at both 9 and 18 day time points. Hypoxia responsive *dosR* (Rv3133c) regulator was also found to be up-regulated at both 9 and 18 days under multiple-stress and the induction level was lower compared to the other dormancy responsive genes tested (Fig. 9); a similar expression pattern for *dosR* was also observed by DNA-microarray analysis.

### Failure of *tgs1* deletion mutant to develop Rif resistance under multiple stress

**Figure 10:**
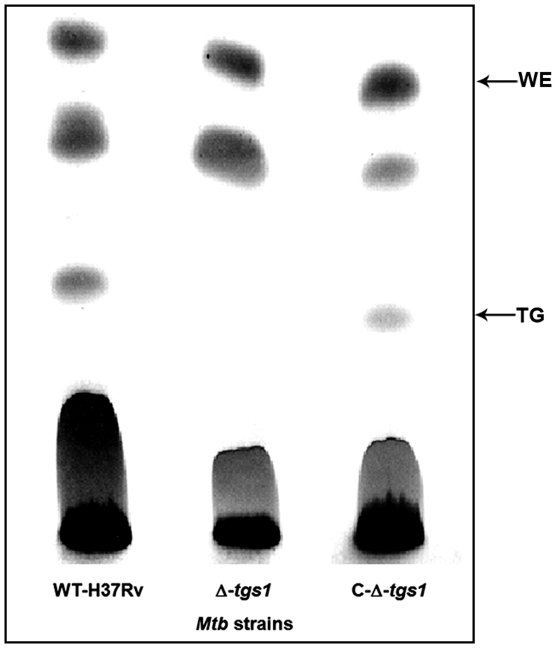
Loss of TG accumulation in Δ-*tgs1* (Δ-Rv3130c) under multiple-stress (18 days) and its restoration by complementation. Equal amount of lipid for each strain was loaded to Silica-TLC and resolved using hexane-diethyl ether-formic acid (90∶10∶1, v/v/v) solvent system. Lipids were visualized by charring for 10 min at 180°C after spraying with dichromate-sulfuric acid. WE, wax ester; TG, triglyceride; WT-H37Rv, *Mtb* H37Rv; Δ-*tgs*1, *tgs1* deletion mutant of *Mtb* H37Rv; C-Δ-*tgs*1, *tgs1* complemented strain of Δ-*tgs*1 mutant.

The *tgs1* deletion mutant failed to accumulate TG when subjected to multiple-stress condition, while complementation of this mutant with *tgs1* restored the ability to store TG under the same multiple-stress treatment (Fig. 10). Interestingly, *tgs1* mutant failed to develop resistance against Rif and INH to the extent as the wild type (H37Rv) under multiple-stress, whereas the ability to develop Rif resistance was restored by complementation of the mutant with *tgs1* (Table 2).

**Figure 11:**
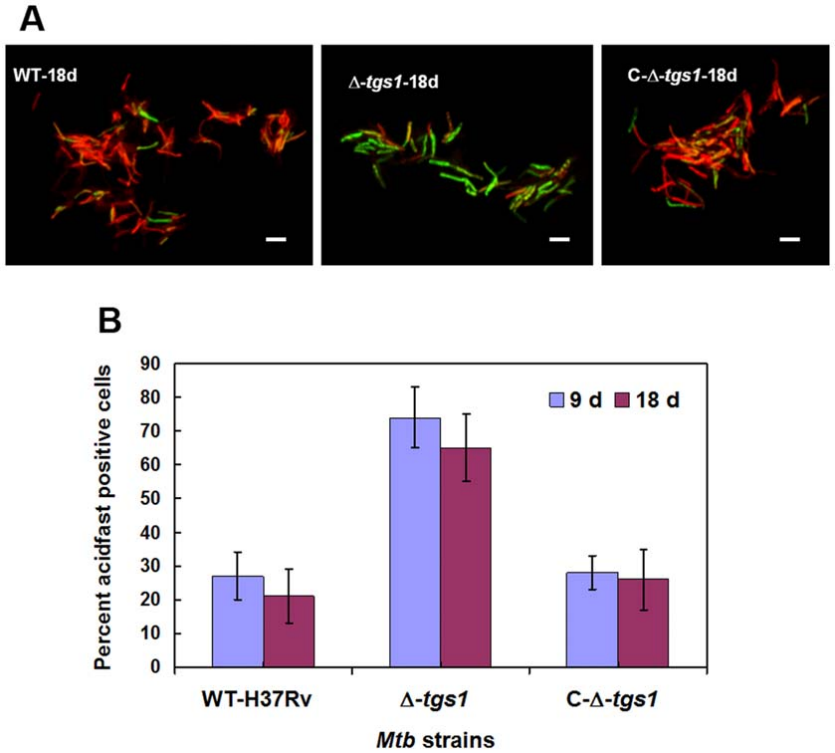
(A), Diminished ‘loss of acid-fastness’ in the Δ-*tgs1* mutant population under multiple-stress. WT-H37Rv, Δ-*tgs1* mutant and C-Δ-*tgs1* cells were stained with Auramine-O (acid-fast) and Nile Red (neutral lipid) after 18 days under multiple-stress. Bar = 4 µM. (B), Percent acid-fast stain positive cells observed in different *Mtb* strains under multiple-stress for 9 and 18 days. WT-H37Rv, wild type *Mtb* H37RV; Δ-*tgs1*, *tgs1* deletion mutant of WT-H37Rv; C-Δ-*tgs1*, *tgs1* complemented strain of Δ-*tgs1*.

A higher percentage of the Δ-*tgs1* cells were found to be acid-fast positive compared to the wild type *Mtb* strain (Fig. 11A). The number of acid-fast negative cells developed in the complemented strain (C-Δ-*tgs1*) of Δ-*tgs1* mutant was comparable to the wild type (Fig. 11A). After 18 days under multiple-stress about 70% of the Δ-*tgs1* mutant population was found to be acid-fast staining positive, whereas in the wild type *Mtb* and the C-Δ-*tgs1* population only about 30% of the cells were acid-fast positive (Fig. 11B).

### Adaptation of multiple-stress model for drug screening

In order to assess whether our *in vitro* dormancy model can be used for drug screening, we used a modification of the Alamar Blue dye method [40]–[42] that can allow quick screening for viability of mycobacterial cells following antibiotic treatment [43]. We used the Alamar Blue dye to quantitate the phenotypic resistance of the 9-day multiple-stressed *Mtb* cultures to Rif and INH. Following antibiotic treatment, the culture was diluted into fresh medium without antibiotic to allow the phenotypically drug-resistant viable *Mtb* cells to resume growth. The Rif- and INH-resistance of the same culture were also evaluated by the agar plating method for comparison. As shown in Table 3, the Alamar Blue dye method yielded antibiotic-resistance values that broadly correlated with the resistance values estimated by the agar plating method. Thus, the Alamar Blue dye method can be used with the multiple-stress model to screen chemical libraries to detect compounds that show lethal activity against dormant bacilli. This method is adaptable to high throughput screening.

**Table 3:** Comparison of Antibiotic Resistance Evaluation by Alamar Blue and Agar Plating Methods.

Antibiotic	Percent Antibiotic Resistance in *Mtb* population after day 9 under multiple-stress	
	Alamar Blue	Agar Plating Method
Rif, 0.1 µg/ml	65±8	100
Rif, 5.0 µg/ml	3±1	4±1
INH, 0.8 µg/ml	64±14	40±20

Cultures of *Mtb* at 9 day in multiple-stress were treated with antibiotic for 5 days. Viable cells surviving the antibiotic treatment were allowed to grow for 5 days by 200-fold dilution into media without antibiotic. The viable cells were quantified using Alamar Blue dye as described in Materials and Methods.

## Discussion

The nature of the host environment that causes *Mtb* to go into a latent state is poorly understood. However, reduced oxygen tension, nutrient limitation (carbon and nitrogen), acidic pH, and high carbon dioxide are among the major stress-factors that were thought to be encountered by the pathogen *in vivo* besides the immunological factors [10], [29], [31], [33], [34], [44], [45]. Stresses have been applied to *Mtb* in attempts to generate a dormancy-like state *in vitro* [21], [22], [24], [27]–[29]. *Mtb* that is dormant *in vivo* does not show acid-fast staining, contains lipid inclusion bodies and shows resistance to drug such as INH and Rif. Available data show that *in vitro* application of individual or dual stress conditions to *Mtb* does not cause the pathogen to acquire all of these characteristics. Therefore, we attempted to mimic the *in vivo* conditions by applying a combination of four major stresses comprising of low oxygen tension (5%), high concentration of CO_2_ (10%), low carbon and nitrogen nutrient and acidic pH (5.0). Results presented here show that under this multiple-stress condition *in vitro Mtb* cells acquire all of the major characteristics of *in vivo* dormancy.

Under the multiple-stress condition two types of storage lipids (TG and WE) accumulated in *Mtb* cells. Chromatographic analysis and labeling studies with [^14^C]oleic acid documented accumulation of these storage lipids. The major fatty-acid constituents of TG were found to be palmitate (C_16:0_) and stearate (C_18:0_) under this multiple-stress condition. However, we have previously reported C_26:0_ as the major fatty acid constituent of TG that accumulated in *Mtb* under hypoxic or NO stresses [12], [14]. Under those stress conditions of hypoxia or NO-treatment the media used were not nutritionally poor and we detected upregulation of multifunctional fatty-acid synthase (*fas/*Rv2524c) gene in *Mtb*. C_26:0_ is a major fatty acid generated by this enzyme [46]. However, under the multiple-stress condition, where the medium is nutritionally poor, there was no induction of the *fas* gene and this could be the probable reason why we did not find C_26:0_ fatty-acid as a major constituent of the storage lipids. Presumably these lipid reserves serve as the energy sources for long term survival of *Mtb* in latency [12]–[14], [35], [47]. We have previously shown that TG inside *Mtb* cells is hydrolyzed under nutrient starvation [13] and *Mycobacterium bovis* BCG was reported to preferentially use TG within macrophages [48], indicating that TG is probably used as an energy source by *Mtb* during the course of the disease.

Concomitant with the progressive increase in quantity of storage lipids, as detected by TLC analysis, most of the *Mtb* cells were observed to be loaded with Nile Red-staining lipid droplets by 18 days under multiple-stress. Previously Sudan Black B staining lipid bodies have been found in non-dividing bacilli [49]. Nile Red-staining lipid droplets were found in *Mtb* cells from sputum samples and these lipid-loaded cells from human patients were found to be dormant [35], [36]. The appearance of the lipid loaded *Mtb* cells from the multiple-stress *in vitro* model of dormancy was very similar to that of the dormant *Mtb* cells from human patients [35].

Loss of acid-fastness seems to be an important trait for non-replicating dormant cells from *in vitro* cultures or from tuberculous lesions and most of such cells would grow under favorable growth conditions with restoration of acid-fastness [50]–[53]. We have also observed restoration of acid-fastness, when the dormant cells generated under multiple-stress were grown in fresh media with complete nutrients (data not shown). Under the multiple-stress, nearly all of the *Mtb* cells with lipid droplets failed to show acid-fast staining. Acid-fast staining is usually performed by conventional Ziehl-Neelson method to diagnose active TB using sputum samples. But in the heterogeneous population of *Mtb* from sputum samples many of the lipid loaded cells were lacking Auramine-O (acid-fast) stain as well [36]. Development of INH resistance has been shown to be associated with loss of acid-fastness in a *kasB* deletion mutant of *Mtb*, where loss of acid-fastness was found to be due to the lack of mycolic acid biosynthesis [54]. Microarray analysis showed that under multiple-stress *kasB* was down-regulated in *Mtb*. Thus, under the multiple-stress, *Mtb* cells become non-replicative and they lose acid-fast staining property probably due to the shut down of mycolic acid synthesis and thus exhibit INH-tolerance at a higher frequency and at a earlier time point as compared to the development of Rif tolerance [55]. The possible connection between INH resistance phenotype and loss of acid-fastness owing to the absence of mycolic acid [54] was also supported by the reduced ‘loss of acid-fast’ staining property and higher degree of INH killing in the Δ-*tgs1* mutant as compared to the wild type and the *tgs1* complemented strain.

Drug tolerance is a characteristic feature of dormancy *in vivo* [4]. However, development of drug tolerance has been assessed only in few *in vitro* dormancy models [21], [24]. Significant resistance to moderate levels of Rif (5 µg/ml) has not been achieved within a fairly short time in any of the *in vitro* models tested previously. In the widely used Wayne model of dormancy based on hypoxia, the maximum phenotypic Rif-resistance obtained against a very low concentration of Rif (0.1 µg/ml) was about 21% after 8 days and gradually decreased to 17% tolerance after 14 days and to 12% after 22 days [24]. In the present multiple stress model, by 9 days under stress 100% of the cells were resistant to 0.1 µg/ml of Rif (data not shown). Under the multiple-stress condition the Rif-resistance against a 50 times higher concentration of Rif (5 µg/ml) increased gradually to approximately 5% and 12% within 9 and 18 days respectively. In the nutrient starvation model about 60% tolerance against Rif (at 1 µg/ml) was reported after 42 days under the stress [21], whereas, in our model we observed about 50% survival at the same concentration of Rif within 18 days (data not shown). Percent resistance against INH is generally higher compared to Rif in the same bacterial population under stress, as INH can kill only actively dividing cells whereas Rif can kill growing as well as non-dividing cells with short spurts of metabolism [4]. Development of a higher frequency of Rif-resistance against a higher concentration of the drug indicates the development of true dormancy of *Mtb* under multiple-stress. The model suggests a link between the occurrence of higher phenotypic drug resistance and storage lipid accumulation. Under the multiple-stress condition no TG could be detected in the *tgs1* deletion mutant. The *tgs1* deletion mutant with compromised accumulation of TG was not able to develop phenotypic antibiotic-resistance comparable to that reached by the wild type. The linkage between lipid accumulation and Rif resistance was strongly supported by the finding that complementation of *tgs1* mutant restored development of Rif resistance. Moreover, the higher percentage of acid-fast positive cells in *tgs1* mutant, with impaired TG accumulation ability under multiple-stress, suggests links between loss of acid-fastness, lipid accumulation and development of phenotypic antibiotic resistance during the development of true dormancy. The molecular basis of these linkages remains to be elucidated.

We investigated the gene regulation reflecting overall metabolic and physiological changes that occur when *Mtb* is subjected to the multiple stress condition by measuring the gene transcript levels at different time points under multiple-stress using whole genome microarray and qRT-PCR. Several TG and WE biosynthetic genes were up-regulated. Induction level of *tgs1* (Rv3130c) was the highest among the *tgs* genes measured by qRT-PCR, which is comparable to our previous reports where *tgs1* was the maximally expressed gene under different stress conditions [12]. TGS1 was also the most active enzyme when expressed in *E. coli* [12]. Reports on the use of many other stress conditions on *Mtb* have also shown induction of *tgs1*, which is one of the genes of the hypoxia responsive dormancy regulon [12], [45], [56]–[59]. We identified *tgs1* as the critical gene for the biosynthesis of TG in the bacterium under *in vitro* dormancy-like conditions induced by single stress factors [14]. It has been suggested that constitutive accumulation of TG by the W/Beijing strains may confer an adaptive advantage for growth and survival in microaerophilic or anaerobic environments and thus be related to the strength of epidemiological spread by this strain [47]. Besides *tgs1* (Rv3130c), Rv3371 and Rv1760 were significantly up-regulated as detected by qRT-PCR and 10 other *tgs* genes were also induced at a lower level. Induction of Rv3371 and Rv1760 increased gradually with time. Our microarray analysis of transcripts also revealed that Rv3371 was significantly up-regulated *tgs* gene under multiple-stress condition. This *tgs* gene was also shown to be up-regulated in human lung granuloma and in pericavity [60]. Rv3734c (*tgs2*) was reported to be induced under nutrient starvation [21]. Induced expressions of Rv3088 (*tgs4*) and Rv3087 under multiple-stress are worth noting as these two genes were previously found to be induced by acidic shock and these are the only two *tgs* genes that belong to a putative acid inducible *mym*A operon [61] and were also reported to be up-regulated under nutrient starvation [21]. Rv0221 and Rv1425 were reported to be up-regulated in intraphagosomal lesions [58]. We previously detected significant upregulation of several *tgs* genes under hypoxic and NO exposures, particularly for those which exhibited highest TG synthase activity when expressed in *E. coli* [12].

It is noteworthy that according to a recent report on meta-analysis of *Mtb* transcription profile data obtained using various *in vitro* and *in vivo* stress conditions, *tgs1* (Rv3130c), Rv3371 and Rv1760 had the highest up-regulation scores among the *tgs* genes [62]; and were placed in the same order of ranking based on induction levels that we measured under multiple-stress condition. Many other *tgs* genes which were reported with up-regulations scores in the meta-analyses [62], were also found to be induced under the multiple-stress condition. The meta-analysis of *Mtb* gene expression data also reported upregulation scores for Rv3391 (*fcr1*) and Rv1543 (*fcr2*) that are probably involved in WE biosynthesis. Only *fcr1* (Rv3391) was found to be induced under multiple-stress condition by qRT-PCR, whereas both *fcr1* (Rv3391) and *fcr2* (Rv1543) were detected to be up-regulated by microarray analysis [GEO accession: GSE10391]. *fcr1* (Rv3391) was also reported to be induced after 96 h of nutrient starvation but no induction was reported for the *fcr2* (Rv1543) or *fcr3* (Rv1544) under that starvation condition [21]. Variable expression profile of different *tgs* or *fcr* genes under different stresses raise the possibility that lipid accumulation under different stress conditions might use different sets of *tgs* and *fcr* genes.

Since multiple-stress generates what appears to be truly dormant *Mtb* cells similar to those found *in vivo*, this *in vitro* dormancy model might be suitable for screening of drug candidates that can kill dormant *Mtb*. To use cultures containing the dormant cells for such drug candidates testing we need a convenient method to measure the killing of dormant cells that can be adapted for high-throughput screening. Alamar Blue dye reduction method was used to measure the viable cells remaining after drug treatment. This method yielded values that were comparable to the traditional cfu count method, but the results were obtained in much less time. This method can be adapted for use in high throughput screening of chemical libraries for novel antilatency drug candidates. If new drugs can be developed and used along with the available frontline drugs then TB cure can be achieved in a short period and such an approach could lead to eventual eradication of TB. This is the first report of an *in vitro* multiple-stress dormancy model for *Mtb* that manifests all features characteristic of *in vivo* dormancy and implicate a critical link between storage lipid accumulation and drug-tolerance.

## Materials and Methods

### Strains, Stock culture and Development of multiple-stress *in vitro* dormancy model

*Mycobacterium tuberculosis* H37Rv (*Mtb*), *tgs*1 (Rv3130c) deletion mutant of *Mtb* (Δ-*tgs1*) and *tgs1* complemented strain of Δ-*tgs*1 (C-Δ-*tgs*1) were grown in Middlebrook 7H9 broth supplemented with 0.2% glycerol and 10% Middlebrook OADC enrichment (Difco) up to OD_600 nm_ of 0.7, mixed with glycerol to a final concentration of 15%, and stored at -80.0°C as stock cultures before they were used to inoculate complete Dubos medium to prepare culture for multiple-stress application. Δ-*tgs*1 and C-Δ-*tgs*1 strains were grown in media containing hygromycin (Hyg, 75 µg/ml) and Hyg (75 µg/ml) plus kanamycin (Kan, 30 µg/ml) antibiotics respectively.

*Mtb* strains from frozen stock were grown in complete Dubos (Difco) medium containing 1.5% glycerol and 10% Dubos-medium-albumin-supplement to an OD_600 nm_ of 0.2 in a roller bottle incubator at 37°C. This seed culture was used to inoculate a second batch of culture and grown up to an OD_600 nm_ of 0.2. Cells were harvested and resuspended to obtain OD_600 nm_ of 0.2 in an acidic (pH 5.0), low nutrient Dubos medium (10% of Dubos medium with Dubos albumin supplement and without glycerol) supplemented with 0.018% Tyloxapol surfactant. 150 ml of this cell suspension was placed in a 1000 ml glass bottle fitted with a 24/40 standard joint neck, and the bottle was sealed with a tight-fitting rubber septum flip top cap. Molten paraffin wax was layered on the out side of the cap to ensure air-tight seal. Each bottle was flushed using 18G needle attached to a 0.2 µm filter with a gas mixture (5% O_2_+10% CO_2_+85% N_2_) at the rate of 1000 ppm for 5 min. Oxygen consumption was measured on a daily basis and only a slight oxygen depletion could be detected after 3 days. Therefore, the bottles were flushed with the gas mixture on every alternate day. Sealed culture bottles were rolled in a roller incubator at 37°C. Cells from a set of bottles were harvested at different (0 to 18 days) time points and were stored at −80°C until used to isolate RNA to determine gene expression profile by microarray and quantitative RT-PCR, and to perform lipid analysis by TLC. Aliquots of 2 ml cultures were treated with respective antibiotics to determine phenotypic antibiotic resistance by plate count method (cfu counting).

### Lipid analysis of *Mtb* cells treated with multiple-stress condition

*Mtb* cultures were subjected to the multiple-stress condition for 0, 3, 9 or 18 days and total lipids were extracted and analyzed by silica-thin layer chromatography (TLC) as described previously [12]. For metabolic incorporation studies *Mtb* cells under multiple-stress condition were incubated with [1-^14^C]oleic acid and the lipids were extracted and analyzed as previously described [12]. Wax ester (WE), and triacylglycerol (TG) fractions were purified from preparative silica-TLC plates and the constituent fatty acids were converted to methyl esters by BF_3_/methanol transesterification. The fatty acid methyl esters were analyzed by capillary gas chromatography using a Varian CP-TAP CB column (25 m×0.25 mm×0.1 µm; He 2.2 ml/min) attached to a Varian CP-3900 gas chromatograph under a temperature control program (70°C, 0.1 min; 40°C/min to 280°C; 4°C/min to 320°C).

### Auramine-O/Nile Red dual fluorescent staining to determine loss of acid-fastness and accumulated lipid bodies

Fluorescent acid-fast staining dye Auramine-O was used in combination with neutral lipid staining dye Nile Red (9-Diethylamino-5H-benzo-α-phenoxazine-5-one) using a modified method described by Garton *et al* [36]. About 20 µl of culture at different time points were evenly spread to make a thin smear on a glass slide, quickly heat fixed on a flame and cooled to room temperature before staining. Each smear was covered with Auramine-O (10 µg/ml), incubated for 20 min, gently washed with distilled water, treated with decolorizer solution for 30 sec, washed with distilled water, covered with the second dye Nile Red solution (10 µg/ml in ethanol), incubated for 15 min, washed with water, covered with potassium permanganate solution for 1 min, washed thoroughly with water and air dried. The staining procedure was carried out in BSL3 hood without direct light exposure. Stained smears were mounted using Cytoseal60 and thin cover glass (less than 150 micron thick). The mounted slides were dried under darkness for at least 8 hrs before examining under fluorescent (Nikon) and confocal laser scanning microscopes (Leica TCS SP5). The confocal scanning images were analyzed and projected using the LAS AF software for Leica TCS SP5 confocla systems. The stability of both the dyes were tested using different mounting media for different time periods and no significant loss of fluorescence intensity due to quenching or leaching of the dyes was observed with the mounting medium used.

### Fractionation of mycobacterial cells by density gradient centrifugation

We tested a self-generated gradient based on a starting density (ρ of 1.07 g/ml), which would cover a range of ρ from ∼1.13 to 1.01 g/ml. Following manufacturer's protocol (GE Healthcare, USA), we made a Stock Isotonic Percoll (SIP) solution by mixing 9 parts Percoll with 1 part sterile 1.5 M NaCl. The SIP was further diluted to ρ = 1.07 g/ml with sterile 0.15 M NaCl. To form the gradient, 9.5 ml of the 1.07 ρ solution was pipetted into 10 ml Seton Easy-Seal polyallomer centrifuge tubes with the Seton Noryl crown assembly and centrifuged without brakes in a Beckman Optima L-90K ultracentrifuge at 18,000 rpm at 20°C for 20 min, using a Ti 70.1 rotor. *Mtb* cell sample at different time points were centrifuged and resuspended in complete 7H9 medium to give one OD_600_ equivalent per ml. One ml of such *M. tuberculosis* cell suspension was carefully layered on top of the gradient. All relevant steps are carried out under aseptic conditions. The tubes were centrifuged at 400 g for 16 min in a Sorvall Legend RT clinical centrifuge with a swinging bucket rotor at 5°C.

### Phenotypic antibiotic resistance by plate count method (cfu counting)

The phenotypic antibiotic resistance against different concentrations of rifampicin (Rif) and isoniazid (INH) was measured by plate count method (cfu counting). At different time points under multiple-stress condition 2 ml aliquots of the cultures were placed in 25 ml glass tubes, to each tube appropriate aliquot of an antibiotic was added, sealed tightly with septum rubber caps and flushed with the same multiple-stress gas mix before incubating at 37°C for 5 days. After incubating for 5 days under antibiotic treatment, serial dilutions were made in Middlebrook 7H9 liquid medium and appropriate dilutions were spread on Middlebrrok 7H10 agar plates without any antibiotic. Colonies were counted after 4 weeks of incubation at 37°C. A tube which did not receive any antibiotic was used as a control to determine the total cfu present.

### Quantitation of phenotypic drug resistance using Alamar Blue dye

Aliquots of *Mtb* culture after multiple-stress treatment for 9 days were incubated with Rif (5 µg/ml) for 5 days at 37°C as stated above. The cultures were then diluted 1000-fold into Middlebrook 7H9 medium containing no antibiotic and incubated in a roller incubator at 37°C for 5 days to allow the viable cells to grow. Alamar Blue dye (diluted 100-fold) was added and the increase in fluorescence at 590 nm, after excitation at 530 nm, was monitored at 0, 4, 8 and 24 h after addition of the dye using a BioScan Chameleon V plate reader. Fluorescence readings above the autofluorescence controls were calculated and used to quantitate phenotypic resistance to the antibiotics by comparison with *Mtb* culture not treated with any antibiotic.

### Microarray hybridization, data processing and functional clustering of genes

The whole genome microarray (PFGRC, http://pfgrc.jcvi.org) consisted of 70-mer oligonucleotides for each ORF representing 4,127 ORFs from *M. tuberculosis* strain H37Rv, and 623 unique ORFs from *Mtb* strain CDC 1551 which are not present in the H37Rv strain's annotated gene complement (98% of H37Rv ORFs). The full 70-mer complement was printed in replicate of four spots on the surface of a microarray slide. Total Mtb RNA was isolated using a TRIzol (Invitrogen) extraction and RNeasy (Qiagen) purification as described [29]. A two-color (Cyanine 3 (Cy3) and Cyanine 5 (Cy5)) hybridization format was used for the microarray analysis. Generally, RNA extracted from cells growing exponentially at an optical density of 0.3 in Dubos (pH 7.0), was used to create fluorescent Cy3-labeled reference cDNA for each experiment. The reference cDNA was hybridized together with the Cy5-labeled cDNA synthesized from RNA extracted from cells grown under experimental multiple-stress conditions. All hybridizations were performed with dye-reversal replicates. Labeling cDNA and hybridization were conducted following instructions from PFGRC, JCVI (http://pfgrc.jcvi.org/index.php/microarray/protocols.html). QuantArray (ver. 3.0, Perkin Elmer) was used for 16-bit TIFF image quantification and initial data visualization. The hybridization signal was subjected to normalization and clustering by using open-source R (ver. 2.1.1) packages and S-Plus (Insightful, WA). Intensity-dependent print-tip MAD normalization, Quantile normalization, and hierarchical clustering [63] were performed with R as described before [64]. Significantly expressed genes (a fold change of >2.0) were identified by two different; Benjamini-Hochberg multiple testing correction-ANOVA-test (α = 0.05) [65], and the two-class unpaired algorithm from the Significance Analysis of Microarrays package (SAM, http://www-stat.stanford.edu/~tibs/SAM/). In SAM analysis, we chose the delta value such that the median false discovery rate was less than 1% [37]. ORFs were identified based on the annotation of TubercuList Database (http://genolist.pasteur.fr/TubercuList/index.html).

Genes induced by multiple-stress condition were clustered by their annotated functions in response to three different stresses reported previously (nutrient starvation [21], acidic shock [22] and hypoxic treatment [39]). Data from previous reports of independent microarray analysis of gene transcripts were referred to classify unknown hypothetical proteins. Gene transcription profiles of cells that were treated only under nutrient starvation (10% Dubos medium-without glycerol at pH 7.0), was used as the internal control.

#### Microarray data compliance and accession number

The microarray data presented and discussed in this article are in accordance with MIAME guidelines, deposited in Gene Expression Omnibus of NCBI (GEO [http://www.ncbi.nlm.nih.gov/geo/]) and are accessible through GEO series accession number: GSE10391.

### RNA isolation and real-time Taqman RT-PCR (qRT-PCR)

To each cell pellet from 50 ml culture of untreated and multiple-stress treated cells at different time points, 1 ml Trizol (Invitrogen) containing 20 µg/ml linear acrylamide was added and resuspended by pipetting. The cell suspension was transferred to a 2 ml screw cap tube (Lysing matrix B, Q-Biogene) containing 0.5 ml of 0.1 mm zirconia/silicon beads, disrupted thrice for 40 sec each at speed 6 (Fast Prep cell disruptor, Q-Biogene) with cooling on ice-water for 1 min after each cycle of burst. The disrupted cell suspension was centrifuged for 5 min at high speed (Eppendorf, 5415D) and the supernatant was transferred to a 2 ml snap cap tube containing heavy phase lock gel (5 Prime, Fisher Scientific Co.). 300 µl chloroform was added to each tube, vortexed at high speed for 15 sec, inverted vigorously for 2 mins and centrifuged for 10 min at maximum speed (Eppendorf 5415D). The upper aqueous phase was transferred to a 2.0 ml tube, disinfected with Vesphene-II and taken out from BSL-3 for further processing and maintained on ice until RNA was precipitated on the same day. To this aqueous suspension Glycoblue coprecipitant (100 µg/ml; Ambion Cat#9515), 1/10 volume of 5 M ammonium acetate (Ambion) and equal volume of isopropanol were added. Tubes were incubated at −20°C for 2 hr and centrifuged for 30 min at 4°C (Sorvall). Supernatant was discarded, the RNA pellet was washed twice in 70% ethanol and air dried (<5 min) after removing all the liquid droplets by pipetting. RNA was suspended in appropriate volume of nuclease free water (Ambion), linear acrylamide was added to a final concentration of 20 µg/ml and purified using RNeasy mini column following the manufacturer's instructions. Column purified RNA was treated with RQ1 DNase (Promega) for 30 min at 37°C and purified by RNeasy mini column (Qiagen, USA) following the manufacturers' instructions. Equal amount of RNA from each sample was used to synthesize cDNA using superscript-III reverse transcriptase (Invitrogen, USA), Ribolock RNase inhibitor (Farmentas, USA) and random hexamers following manufacturer's instruction (Invitrogen). cDNA was used at an appropriate dilution for real-time Taqman RT-PCR amplification. All the PCR primer and Taqman probes (5′-6-FAM reporter and 3′-BHQ1 quencher) were designed by using Primer Express software (v 3.0, Applied Biosystems Inc.) and 2× Taqman Fast Universal PCR master mix reagent (Applied Biosystems Inc., USA) was used for PCR amplification and quantification with 7500 Fast real-time PCR system (Applied Biosystems Inc., USA). Default real-time cycling parameter (1 cycle at 95.0°C for 20 sec followed by 40 cycles of 95.0°C for 3 sec and 60°C for 30 sec) and ramp rate for Taqman PCR of 7500 Fast SDS system was used. Data was analyzed by SDS v1.4 software using relative quantification module (ddCt method) to obtain the fold change values for each target gene with the *sigA* as the endogenous control for normalization and day 0 transcript level for each target gene as the calibrator.
